# Supplementary material for: A neurodevelopmental epigenetic programme mediated by SMARCD3–DAB1–Reelin signalling is hijacked to promote medulloblastoma metastasis
Source: Nat Cell Biol. 2023 Feb 27;25(3):493–507. doi: 10.1038/s41556-023-01093-0 (PMC10014585; doi:10.1038/s41556-023-01093-0)
Supplement: Supplementary file 1 — Supplementary Figs. 1–3. [file 41556_2023_1093_MOESM1_ESM.pdf]

nature cell biology

Article https://doi.org/10.1038/s41556-023-01093-0

A neurodevelopmental epigenetic programme mediated by SMARCD3–DAB1–Reelin signalling is hijacked to promote medulloblastoma metastasis

In the format provided by the authors and unedited

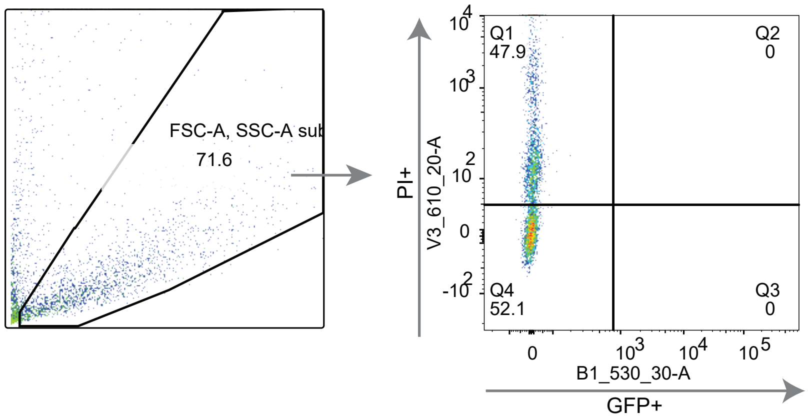

Supplementary Figure 1. **Flow cytometry gating for propidium iodide (PI) positive**. MED8A cells were treated by Hydrogen Peroxide (H2O2) and then analyzed by flow cytometry.

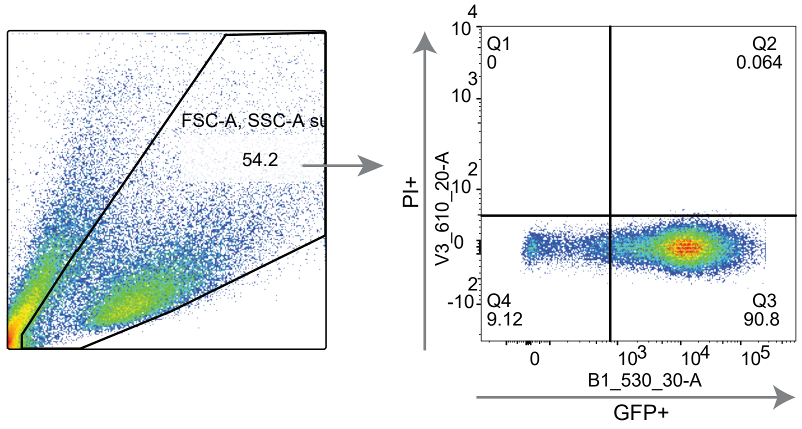

Supplementary Figure 2. **Flow cytometry gating for GFP positive**. MED8A cells were labeled by lentivirus-mediated GFP expression and then analyzed by flow cytometry.

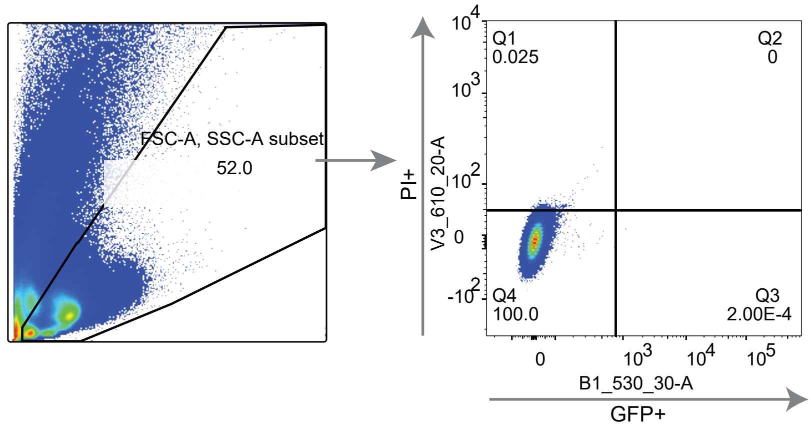

Supplementary Figure 3. **Flow cytometry gating for PI and GFP negative**. Mouse PBMCs were isolated and treated with RBC lysis buffer. Then, cells were suspended in ice-cold PBS with 1% BSA and 2mM EDTA for flow cytometry analysis.
