# Supplementary material for: A neurodevelopmental epigenetic programme mediated by SMARCD3–DAB1–Reelin signalling is hijacked to promote medulloblastoma metastasis
Source: Nat Cell Biol. 2023 Feb 27;25(3):493–507. doi: 10.1038/s41556-023-01093-0 (PMC10014585; doi:10.1038/s41556-023-01093-0)
Supplement: Supplementary file 2 — Reporting Summary [file 41556_2023_1093_MOESM2_ESM.pdf]

nature portfolio

Corresponding author(s): Sarah J. Hainer, Michael D. Taylor, Baoli Hu

Last updated by author(s): Jan 13, 2023

Reporting Summary

Nature Portfolio wishes to improve the reproducibility of the work that we publish. This form provides structure for consistency and transparency in reporting. For further information on Nature Portfolio policies, see our Editorial Policies and the Editorial Policy Checklist.

Statistics

For all statistical analyses, confirm that the following items are present in the figure legend, table legend, main text, or Methods section.

n/a	Confirmed	
☐	☒	The exact sample size (*n*) for each experimental group/condition, given as a discrete number and unit of measurement
☐	☒	A statement on whether measurements were taken from distinct samples or whether the same sample was measured repeatedly
☐	☒	The statistical test(s) used AND whether they are one- or two-sided<br>*Only common tests should be described solely by name; describe more complex techniques in the Methods section.*
☐	☒	A description of all covariates tested
☐	☒	A description of any assumptions or corrections, such as tests of normality and adjustment for multiple comparisons
☐	☒	A full description of the statistical parameters including central tendency (e.g. means) or other basic estimates (e.g. regression coefficient) AND variation (e.g. standard deviation) or associated estimates of uncertainty (e.g. confidence intervals)
☐	☒	For null hypothesis testing, the test statistic (e.g. *F*, *t*, *r*) with confidence intervals, effect sizes, degrees of freedom and *P* value noted<br>*Give P values as exact values whenever suitable.*
☒	☐	For Bayesian analysis, information on the choice of priors and Markov chain Monte Carlo settings
☒	☐	For hierarchical and complex designs, identification of the appropriate level for tests and full reporting of outcomes
☐	☒	Estimates of effect sizes (e.g. Cohen's *d*, Pearson's *r*), indicating how they were calculated

Our web collection on statistics for biologists contains articles on many of the points above.

Software and code

Policy information about availability of computer code

Data collection	The RNAseq data were generated in this study by using Illumina NovaSeq 6000 platform; and the ATACseq and CUT&RUN were generated by using Illumina NextSeq 500 platform. The detailed information was available in Methods.
Data analysis	Hisat2 (v.2.1.0) was used to map reads from RNAseq data.<br>Seurat (v.3.2.3) was used to analyze the scRNAseq data.<br>Cicero (v.1.6.2) was used to analyze the sci-ATAC-seq3 data.<br>bowtie2 (v.2.3.5.1) was used to map reads from ATACseq and CUT&RUN data.<br>macs3 (v.3.0.0a6) was used to define accessible sites of ATACseq and CUT&RUN data.<br>R (v.3.5.1) was used to perform other statistical analysis and graph plots.<br>GSVA (v.1.36.3) was used to calculate the meta-PCNA score.<br>Cellranger (v.5.0.1) was used to analyze the scRNAseq and sci-ATAC-seq3 data.<br>IPA (v.01-16) was used to analyze pathway.<br>ImageJ (Fiji 1.53C) was used to analyze images taking by microscopes.<br>IGV (v.2.6.3) was used to visualize the peaks and alignments.<br>FlowJo (v.10.6.1) was used to analyze flow cytometry data.<br>GraphPad Prism (v.9.1.0) was used to analyze data of wound-healing assay, transwell assay, extent of metastasis, qRT-PCR, and statistical analysis.<br>Detailed information can be found in Methods.

For manuscripts utilizing custom algorithms or software that are central to the research but not yet described in published literature, software must be made available to editors and reviewers. We strongly encourage code deposition in a community repository (e.g. GitHub). See the Nature Portfolio guidelines for submitting code & software for further information.

Data

Policy information about availability of data

All manuscripts must include a data availability statement. This statement should provide the following information, where applicable:

- Accession codes, unique identifiers, or web links for publicly available datasets
- A description of any restrictions on data availability
- For clinical datasets or third party data, please ensure that the statement adheres to our policy

The availability of data is provided in the paper, which includes:
1) The primary datasets: the RNAseq, ATACseq, and CUT&RUN data that were generated in this study were deposited in the Gene Expression Omnibus (GEO) with accession number GSE194217. At this time, the editors and the reviewers can use this token (wnyvqusifjknfyd) to access the data using this link:https://www.ncbi.nlm.nih.gov/geo/query/acc.cgi?acc=GSE194217.
2) The referenced datasets: previously published data that were re-analyzed in the study include: transcriptomics of 1350 MBs and 291 normal cerebellum samples (GSE124814), scRNAseq data of 25 MBs (GSE119926), expression profiles and clinical data of 763 MBs (GSE85217), Hi-C data of mouse cerebellum (GSE138822), scRNAseq data of developing mouse cerebellum (European Nucleotide Archive: PRJEB23051), ChIPseq data of 5 MBs (GSE92585), sci-ATAC-seq3 data of fetal cerebellum (GSE149683), ChIPseq data of D458 and D425 (GSE129521), proteomic data of 45 MBs (Supplemental Table), 167 MB RNAseq data from R2 (https://r2.amc.nl), processed TCGA pan-cancer RNAseq data from Xena (https://xena.ucsc.edu/), gene profiling of normal human tissues from GTEx (https://www.gtexportal.org/home/), human cerebellum scRNAseq data were obtained from the Human Cell Atlas (https://www.covid19cellatlas.org/aldinger20), ChIPseq data of mouse cerebellum from ENCODE portal (https://www.encodeproject.org/), H3K27ac ChIPseq data of 4 MB subgroups from St. Jude Cloud Visualization Community (https://viz.stjude.cloud/).

Human research participants

Policy information about studies involving human research participants and Sex and Gender in Research.

Reporting on sex and gender	We analyzed the clinical data/samples of 10 medulloblastoma patients including 6 male and 4 female subjects from the bio-repositories in the Xiangya Hospital, Central South University. The MB tissue microarray FFPE slides were obtained from the bio-repositories at Johns Hopkins University. The sex and gender of these patients were provided by the bio-repositories, which were determined based on self-reporting and clinical routine physical examination. Our analysis in this study did not find significant differences in sex and gender. This study does not focus on sex and gender differences either.
Population characteristics	All 10 patients (age from 1 to 38 years old) included in this analysis were diagnosed with medulloblastoma based on histological, radiological, and clinical properties. The IMR/CT images and FFPE slides of 10 patients as well as the MB tissue microarray FFPE slides, which are de-identified and de-linked to any subject privacy information, were provided by the bio-repositories.
Recruitment	No patients recruited occurred for this study. Study materials and de-identified medical record information of the 10 medulloblastoma patients and the MB tissue microarray FFPE slides in this study were obtained from the established bio-repositories.
Ethics oversight	Given that the specimens or data were not collected specifically for this study and the subject identifiers linked to these specimens or data were not requested for this study, this study is not considered human subject research. The data and FFPE tissue slides for this study were provided by the bio-repositories that have the IRB-approval protocols, #202110207 (approved by the Clinical Ethics Committee of Xiangya Hospital, Central South University) and #NA_00015113 (approved by the Johns Hopkins University Institutional Review Board). Informed consent was obtained for these bio-repositories. The study is compliant with all ethical regulations.

Note that full information on the approval of the study protocol must also be provided in the manuscript.

Field-specific reporting

Please select the one below that is the best fit for your research. If you are not sure, read the appropriate sections before making your selection.

☒ Life sciences ☐ Behavioural & social sciences ☐ Ecological, evolutionary & environmental sciences

For a reference copy of the document with all sections, see nature.com/documents/nr-reporting-summary-flat.pdf

Life sciences study design

All studies must disclose on these points even when the disclosure is negative.

Sample size	Sample size was chosen on the basis of our previously published studies (PMID: 27863244; PMID: 34228644) and chosen empirically as per the standard custom followed in the field. No statistical method was used to pre-determine sample size.
Data exclusions	We excluded low quality cells during single-cell RNAseq analysis using the criteria as described in the Methods. No other data were excluded.

Replication

For sequencing replication, three replicates were performed for the RNAseq, ATACseq and H3K4me3 in CUT&RUN of SMARCD3 WT and KO in MED8A cells; and two replicates were performed for H3K4me1, H3K27ac and H3K27me3 in CUT&RUN of SMARCD3 WT and KO in MED8A cells.
Number of biological replicates of other experiments were described in figure legends. All attempts of replication were successful.

Randomization

Mice for implanting tumor cells were randomly grouped.
Mice for dasatinib treatment (Figures7j, 7k, and extended data Figures 9f, 9g) were measured for tumor size with IVIS at 7 days after implanting tumor cells, and then were divided into big, middle, or small tumor groups. The mice in each group were randomly grouped into dasatinib standard dose, low dose, or placebo group.
No randomization was performed for other experiments because control groups and treated groups (such as sgRNA knockout, overexpression, and drug treatment what do you mean drug treatment) in these experiments were defined.

Blinding

The investigators were blinded for experimental group during data collection and analysis, IHC or IF analysis for the assessment of protein staining intensity in Figures 1g; 2g, l, m; 4e, 7b-e, and extended data Figures 3a, b, e, g, h, i, k, l; 9b. No blinding was applied for other experiments because the investigators had to know the groups for assessment and analyses.

Reporting for specific materials, systems and methods

We require information from authors about some types of materials, experimental systems and methods used in many studies. Here, indicate whether each material, system or method listed is relevant to your study. If you are not sure if a list item applies to your research, read the appropriate section before selecting a response.

Materials & experimental systems

n/a	Involved in the study
☐	☒ Antibodies
☐	☒ Eukaryotic cell lines
☒	☐ Palaeontology and archaeology
☐	☒ Animals and other organisms
☒	☐ Clinical data
☒	☐ Dual use research of concern

Methods

n/a	Involved in the study
☒	☐ ChIP-seq
☐	☒ Flow cytometry
☐	☒ MRI-based neuroimaging

Antibodies

Antibodies used

The detailed information about all antibodies used in this study was provided below and in Supplementary Table 8.
Rabbit monoclonal anti-SMARCD3/BAF60C (D6F1S), Cat# 62265, RRID:AB_2799624, Cell Signaling Technology
Rabbit polyclonal anti-SMARCD3, Cat# PA5-41093, RRID:AB_2607216, Thermo Fisher Scientific
Goat polyclonal anti-FOXP2 (C terminus), Cat# EB05226, RRID:AB_2107112, Everest
Chicken polyclonal anti-CALB1, Cat# CH22118, RRID:AB_2737107,Neuromics
Rabbit monoclonal anti-SRC (36D10),Cat# 2109, RRID:AB_2106059,Cell Signaling Technology
Rabbit polyclonal anti-Phospho-Src Family (Tyr416),Cat# 2101, RRID:AB_331697,Cell Signaling Technology
Rabbit polyclonal anti-Phospho-Src (Y419),Cat# AF2685, RRID:AB_442167,R&D
Mouse monoclonal anti-b-Actin (Clone AC-74),Cat# A2228, RRID:AB_476697,Sigma-Aldrich
Rabbit recombinant polyclonal anti-H3K4me1,Cat# 710795, RRID: AB_2532764,Thermo Fisher Scientific
Rabbit monoclonal anti-H3K4me3 (clone 15-10C-E4),Cat# 05-745R, RRID: AB_1587134, Millipore
Rabbit polyclonal anti-H3K9me3, Cat# Ab8898, RRID: AB_306848, Abcam
Rabbit polyclonal anti-H3K27ac, Cat# Ab4729, RRID: AB_2118291, Abcam
Rabbit polyclonal anti-H3K27me3,Cat# 07-449, RRID: AB_310624, Millipore
Mouse monoclonal anti-Nestin (Clone rat-401), Cat# MAB353, RRID:AB_94911, Millipore
Rabbit polyclonal anti-GFAP, Cat# Z0334, RRID:AB_10013382, Agilent
Rabbit polyclonal anti-Olig-2, Cat# AB9610, RRID:AB_570666, Millipore
Mouse monoclonal anti-NPR-C (clone E-5), Cat #515449, Santa Cruz Biotechnology
Mouse monoclonal anti-nestin (10c2), Cat# sc-23927, RRID:AB_627994, Santa Cruz Biotechnology
Mouse monoclonal anti-TUBB3 (clone TUJ1), Cat# 801201, RRID:AB_2313773,BioLegend
Mouse monoclonal anti-Synaptophysin (clone SP17), Cat# 837103, RRID:AB_2783410, BioLegend
Rat monoclonal anti-BrdU (clone BU1/75 (ICR1)), Cat# ab6326, RRID:AB_305426, Abcam
Rabbit monoclonal anti-Ki67 (VP-RM04), Cat# VP-RM04 RRID:AB_2336545, Vector Laboratories
Rabbit polyclonal anti-Cleaved Caspase-3 (Asp175) (clone D175), Cat# 9661, RRID:AB_2341188, Cell Signaling Technology
Goat anti-chicken IgY (H+L) secondary antibody, Alexa Fluor™ 647, Cat# A-21449, RRID:AB_2535866, Thermo Fisher Scientific
Donkey anti-rabbit IgG (H+L) highly cross-adsorbed, Alexa Fluor™ 594, Cat# A-21207, RRID:AB_141637, Thermo Fisher Scientific
Labeled polymer-HRP anti-mouse, Cat# K4006, Dako
HRP Horse anti-rabbit IgG polymer reagent, Cat# MP-7401, RRID:AB_2336529, Vector Laboratories

Validation

Regarding SMARCD3 antibody (Cat# 62265,CST), the validation was provided by the the manufacturer's website and also performed by using the MED8A cell line with CRISPR/CAS9 mediated SMARCD3 deletion vs wildtype for IHC and WB assay.

Rabbit polyclonal anti-H3K27me3, Rabbit polyclonal anti-Phospho-Src Family (Tyr416), and Labeled polymer-HRP anti-mouse antibodies have been validated in the following publications: PMID: 32313005 and PMID: 33958790 for Rabbit polyclonal anti-

H3K27me3, PMID: 31263101 and PMID: 29533785 for Rabbit polyclonal anti-Phospho-Src Family (Tyr416); PMID: 27863244 and PMID: 34228644 for Labeled polymer-HRP anti-mouse antibody.

The validations for other antibodies were provided by the manufacturers' websites:
Rabbit polyclonal anti-SMARCD3 https://www.thermofisher.com/antibody/product/BAF60C-Antibody-Polyclonal/PA5-41093
Goat polyclonal anti-FOXP2 (C terminus) https://everestbiotech.com/product/goat-anti-foxp2-c-terminus-antibody/
Chicken polyclonal anti-CALB1 https://www.neuromics.com/CH22118
Rabbit monoclonal anti-SRC (36D10) https://www.cellsignal.com/products/primary-antibodies/src-36d10-rabbit-mab/2109
Rabbit polyclonal anti-Phospho-Src (Y419) https://www.rndsystems.com/products/human-phospho-src-y419-antibody_af2685
Mouse monoclonal anti-b-Actin (Clone AC-74) https://www.sigmaaldrich.com/US/en/product/sigma/a2228
Rabbit recombinant polyclonal anti-H3K4me1 https://www.thermofisher.com/antibody/product/H3K4me1-Antibody-Recombinant-Polyclonal/710795
Rabbit monoclonal anti-H3K4me3 https://www.emdmillipore.com/US/en/product/Anti-trimethyl-Histone-H3-Lys4-Antibody-clone-15-10C-E4-rabbit-monoclonal,MM_NF-05-745R?ReferrerURL=https%3A%2F%2Fwww.google.com%2F
Rabbit polyclonal anti-H3K9me3 https://www.abcam.com/histone-h3-tri-methyl-k9-antibody-chip-grade-ab8898.html
Rabbit polyclonal anti-H3K27ac https://www.abcam.com/histone-h3-acetyl-k27-antibody-chip-grade-ab4729.html

Mouse monoclonal anti-Nestin (Clone rat-401) https://www.emdmillipore.com/US/en/product/Anti-Nestin-Antibody-clone-rat-401,MM_NF-MAB353?ReferrerURL=https%3A%2F%2Fwww.google.com%2F
Rabbit polyclonal anti-GFAP https://www.agilent.com/en/product/immunohistochemistry/antibodies-controls/primary-antibodies/glial-fibrillary-acidic-protein-(concentrate)-76683
Rabbit polyclonal anti-Olig-2 https://www.sigmaaldrich.com/US/en/product/mm/ab9610
Mouse monoclonal anti-NPR-C https://www.scbt.com/p/npr-c-antibody-e-5
Mouse monoclonal anti-nestin (10c2) https://www.scbt.com/p/nestin-antibody-10c2
Mouse monoclonal anti-TUBB3 https://www.biolegend.com/ja-jp/products/purified-anti-tubulin-beta-3-tubb3-antibody-11580
Mouse monoclonal anti-Synaptophysin https://www.biolegend.com/it-it/products/purified-anti-synaptophysin-antibody-16778
Rat monoclonal anti-BrdU https://www.abcam.com/brdu-antibody-bu175-icr1-proliferation-marker-ab6326.html
Rabbit monoclonal anti-Ki67 https://www.labome.com/product/Vector-Laboratories/VP-RM04.html check
Rabbit polyclonal anti-Cleaved Caspase-3 (Asp175) https://www.cellsignal.com/products/primary-antibodies/cleaved-caspase-3-asp175-antibody/9661
Goat anti-chicken IgY (H+L) secondary antibody, Alexa Fluor™ 647 https://www.thermofisher.com/antibody/product/Goat-anti-Chicken-IgY-H-L-Secondary-Antibody-Polyclonal/A-21449
Donkey anti-rabbit IgG (H+L) highly cross-adsorbed, Alexa Fluor™ 594 https://www.thermofisher.com/antibody/product/Donkey-anti-Rabbit-IgG-H-L-Highly-Cross-Adsorbed-Secondary-Antibody-Polyclonal/A-21207
HRP Horse anti-rabbit IgG polymer reagent https://vectorlabs.com/products/enzyme-polymer/immpress-hrp-horse-anti-rabbit-igg

Eukaryotic cell lines

Policy information about cell lines and Sex and Gender in Research

Cell line source(s)	MED8A was provided by Dr. Michael D.Taylor, The Hospital for Sick Children, Toronto, Canada.<br>D556 was provided by Dr. Darell D. Bigner, Duke University Medical Center, Durham, NC.<br>D425 and D458 were provided by Dr. Sameer Agnihotri, UPMC Children's Hospital of Pittsburgh, Pittsburgh, PA.<br>D341 was purchased from ATCC (# HTB-187).<br>The human cerebellar neural stem cells (hcNSCs) was provided by Dr. Eric H. Raabe, Johns Hopkins University School of Medicine, Baltimore, MD.<br>The 293T packaging cells was purchased from ATCC.
Authentication	The MB cell lines used in this study were obtained from the brain tumor labs.<br>The human cerebellar neural stem cells (hcNSCs) was obtained and used in the publication (PMID: 27012813).<br>D341 and 293T cells were purchased from ATCC with the vendor's authentication.<br>These cell lines were not authenticated (such as shot tandem repeat assay) by us in the lab.
Mycoplasma contamination	All cell lines were tested to be negative for mycoplasma using MycoAlert PLUS Mycoplasma Detection Kit (Lonza).
Commonly misidentified lines<br>(See ICLAC register)	No commonly misidentified cell lines from the ICLAC Register were used in the study.

Animals and other research organisms

Policy information about studies involving animals; ARRIVE guidelines recommended for reporting animal research, and Sex and Gender in Research

Laboratory animals	Female and male ICR SCID mice at 4-6 weeks of age were purchased from Taconic Biosciences (Model # ICRS-F/ICRS-M). C57BL/B6 mice 4-6 weeks of age purchased from The Jackson Laboratory (Strain # 000664) were bred and maintained at CHP Rangos Research Center under pathogen-free conditions. All animal experiments were performed with the approval of the University of Pittsburgh Animal Care and Use Committee (IACUC) with #21049271.
Wild animals	The study did not involve wild animals.
Reporting on sex	The study doesn't focus on sex differences. The equal number of female and male SCID mice were used for dasatinib treatment experiments. Female SCID mice were mostly used for tumor xenograft experiments. Both male and female embryos were used to examine SMARCD3 expression experiments (Fig. 4d, e). The difference between female and male mice in the study is not significant.

	Mouse sex was determined through genital area and nipples.
Field-collected samples	The study did not involve samples collected from the field.
Ethics oversight	All animal experiments were performed with the approval of University of Pittsburgh Animal Care and Use Committee (IACUC).

Note that full information on the approval of the study protocol must also be provided in the manuscript.

Flow Cytometry

Plots

Confirm that:

☒ The axis labels state the marker and fluorochrome used (e.g. CD4-FITC).

☒ The axis scales are clearly visible. Include numbers along axes only for bottom left plot of group (a 'group' is an analysis of identical markers).

☒ All plots are contour plots with outliers or pseudocolor plots.

☒ A numerical value for number of cells or percentage (with statistics) is provided.

Methodology

Sample preparation	See Methods, in the section "Flow cytometry and FACS sorting".
Instrument	BD Fortessa and BD FACSAria cell sorter.
Software	Data were analyzed using FlowJo (v.10.6.1).
Cell population abundance	Sorted cells were resorted using the same gating strategy and purity was above 90%.
Gating strategy	For analyzing circulating tumor cells (CTCs), the PBMCs isolated from the mice without tumor cell implantation were used as the negative control, and the GFP-labeled tumor cells were used as the positive control to check the background signal and set the gate for the GFP channel. Tumor cells added hydrogen peroxide or not were used as a positive control or negative control to check the background signal and set the gate for the Propidium iodide (PI) channel.<br>For sorting GFP positive cells, the tumor cells without GFP labeled were used as the negative control to check the background signal and set the gate for cell sorting.

☒ Tick this box to confirm that a figure exemplifying the gating strategy is provided in the Supplementary Information.

Magnetic resonance imaging

Experimental design

Design type	Standard of small animal imaging and standard of care clinical imaging.
Design specifications	Standard design for assessing tumor development and growth in mouse brain. Routine of clinical imaging for human. No specific design was applied.
Behavioral performance measures	Behavioral performance measures were not applicable in the study.

Acquisition

Imaging type(s)	Anatomical
Field strength	7 Tesla for mouse and 3 Tesla for human.
Sequence & imaging parameters	For mouse brain MRI (Extended Data Fig. 3e): T1-weighted with contrast images were obtained with the typical imaging parameters: FOV 3.0 cm × 2.0 cm, acquisition matrix 384 × 256, acquisition slice thickness 0.60 mm, TR/TE = 2177/14 ms.<br>For human brain MRI (Fig. 7a): 2D sagittal T1-weighted FLAIR with contrast images were obtained with imaging parameters: acquisition matrix 320 × 256, acquisition slice thickness 5 mm, TR/TE = 1961/27 ms.
Area of acquisition	Whole brain imaging.

Diffusion MRI ☐ Used ☒ Not used

Preprocessing

Preprocessing software	Preprocessing was not applied in this study.

Normalization	Normalization was not applied in this study.
Normalization template	Normalization template was not applied in this study.
Noise and artifact removal	Noise and artifact removal was not applied in this study.
Volume censoring	Volume censoring was not applied in this study.

Statistical modeling & inference

Model type and settings	No statistical modeling and inference was used in this study.
Effect(s) tested	No statistical modeling and inference was used in this study.

Specify type of analysis: ☒ Whole brain ☐ ROI-based ☐ Both

Statistic type for inference (See Eklund et al. 2016)	No statistical modeling and inference was used in this study.
Correction	No statistical modeling and inference was used in this study.

Models & analysis

n/a	Involved in the study
☒	☐ Functional and/or effective connectivity
☒	☐ Graph analysis
☒	☐ Multivariate modeling or predictive analysis
