# Supplementary figures and images for: A neurodevelopmental epigenetic programme mediated by SMARCD3–DAB1–Reelin signalling is hijacked to promote medulloblastoma metastasis
Source: Nat Cell Biol. 2023 Feb 27;25(3):493–507. doi: 10.1038/s41556-023-01093-0 (PMC10014585; doi:10.1038/s41556-023-01093-0)

**Fig. 1j**

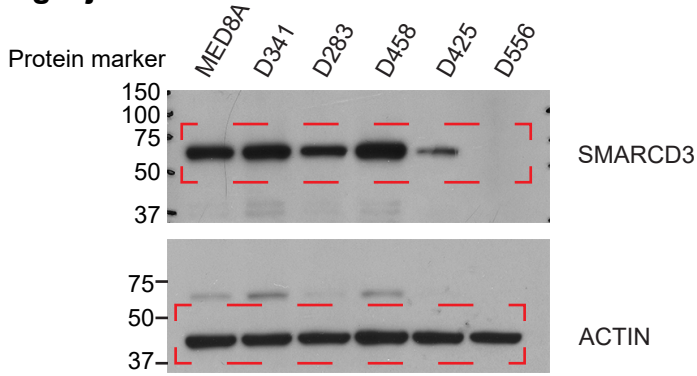

Supplement: Source Data Fig. 1 — Unprocessed western blots and/or gels. [file 41556_2023_1093_MOESM9_ESM.pdf]

Fig. 2a

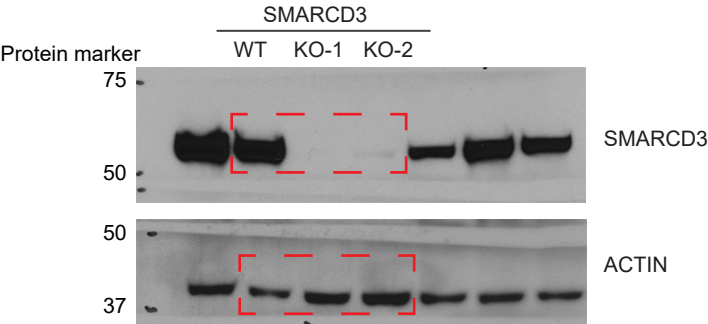

Fig. 2e

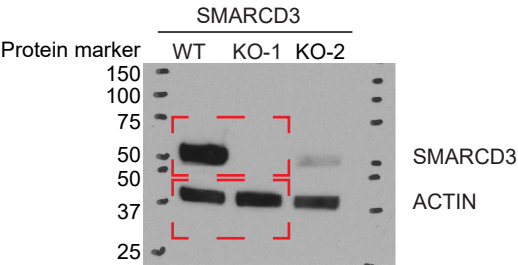

Fig. 2i

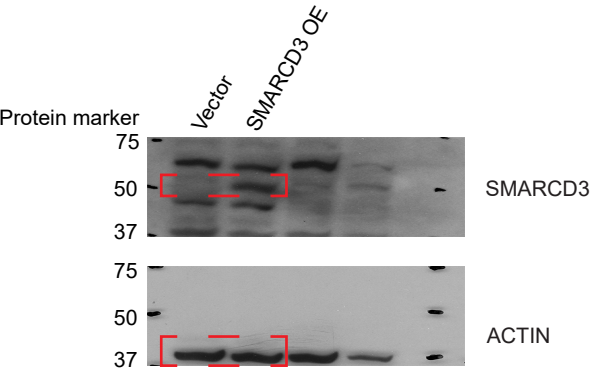

Supplement: Source Data Fig. 2 — Unprocessed western blots and/or gels. [file 41556_2023_1093_MOESM11_ESM.pdf]

**Fig. 7f**

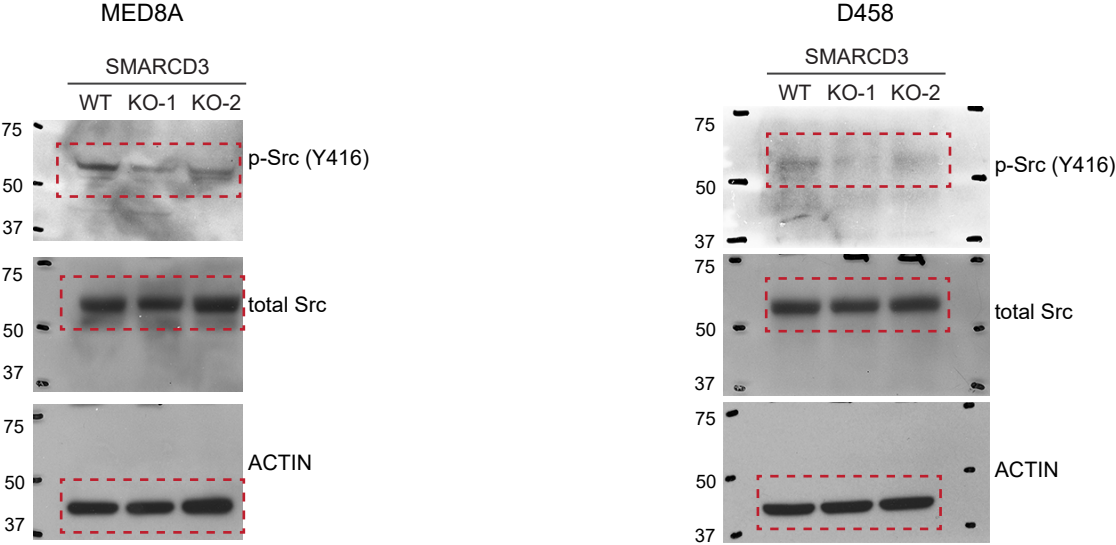

Supplement: Source Data Fig. 7 — Unprocessed western blots and/or gels. [file 41556_2023_1093_MOESM16_ESM.pdf]

Extended Data Fig. 2b

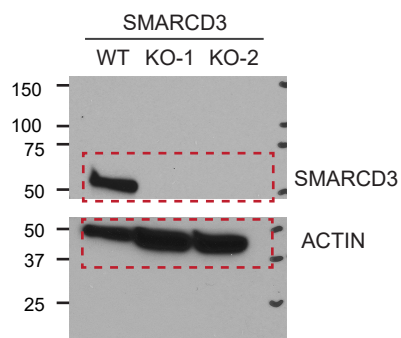

Supplement: Source Data Extended Data Fig. 2 — Unprocessed western blots and/or gels. [file 41556_2023_1093_MOESM19_ESM.pdf]
